# Multiple anthropometric measures and proarrhythmic 12-lead ECG indices: A mendelian randomization study

**DOI:** 10.1371/journal.pmed.1004275

**Published:** 2023-08-08

**Authors:** Maddalena Ardissino, Kiran Haresh Kumar Patel, Bilal Rayes, Rohin K. Reddy, Greg J. Mellor, Fu Siong Ng

**Affiliations:** 1 National Heart and Lung Institute, Imperial College London, London, United Kingdom; 2 Royal Papworth Hospital, Cambridge Biomedical Campus, Cambridge, United Kingdom

## Abstract

**Background:**

Observational studies suggest that electrocardiogram (ECG) indices might be influenced by obesity and other anthropometric measures, though it is difficult to infer causal relationships based on observational data due to risk of residual confounding. We utilized mendelian randomization (MR) to explore causal relevance of multiple anthropometric measures on P-wave duration (PWD), PR interval, QRS duration, and corrected QT interval (QTc).

**Methods and findings:**

Uncorrelated (r^2^ < 0.001) genome-wide significant (*p* < 5 × 10^−8^) single nucleotide polymorphisms (SNPs) were extracted from genome-wide association studies (GWAS) on body mass index (BMI, *n* = 806,834), waist:hip ratio adjusted for BMI (aWHR, *n* = 697,734), height (*n* = 709,594), weight (*n* = 360,116), fat mass (*n* = 354,224), and fat-free mass (*n* = 354,808). Genetic association estimates for the outcomes were extracted from GWAS on PR interval and QRS duration (*n* = 180,574), PWD (*n* = 44,456), and QTc (*n* = 84,630). Data source GWAS studies were performed between 2018 and 2022 in predominantly European ancestry individuals. Inverse-variance weighted MR was used for primary analysis; weighted median MR and MR-Egger were used as sensitivity analyses. Higher genetically predicted BMI was associated with longer PWD (β 5.58; 95%CI [3.66,7.50]; *p* = < 0.001), as was higher fat mass (β 6.62; 95%CI [4.63,8.62]; *p* < 0.001), fat-free mass (β 9.16; 95%CI [6.85,11.47]; *p* < 0.001) height (β 4.23; 95%CI [3.16, 5.31]; *p* < 0.001), and weight (β 8.08; 95%CI [6.19,9.96]; *p* < 0.001). Finally, genetically predicted BMI was associated with longer QTc (β 3.53; 95%CI [2.63,4.43]; *p* < 0.001), driven by both fat mass (β 3.65; 95%CI [2.73,4.57]; *p* < 0.001) and fat-free mass (β 2.08; 95%CI [0.85,3.31]; *p* = 0.001). Additionally, genetically predicted height (β 0.98; 95%CI [0.46,1.50]; *p* < 0.001), weight (β 3.45; 95%CI [2.54,4.36]; *p* < 0.001), and aWHR (β 1.92; 95%CI [0.87,2.97]; *p* = < 0.001) were all associated with longer QTc. The key limitation is that due to insufficient power, we were not able to explore whether a single anthropometric measure is the primary driver of the associations observed.

**Conclusions:**

The results of this study support a causal role of BMI on multiple ECG indices that have previously been associated with atrial and ventricular arrhythmic risk. Importantly, the results identify a role of both fat mass, fat-free mass, and height in this association.

## Abstract 2

In a Mendelian randomization study, Fu Siong Ng and colleagues investigate how different anthropometric measures influence proarrhythmic 12-lead ECG indices.

## Author summary

**Why was the study done?:**

- Previous studies have shown that a higher body mass index (BMI) is associated with changes in electrocardiogram (ECG) measurements.
- However, it is unclear from available research whether this is driven by fat mass or by lean mass because BMI does not differentiate between these.
- Additionally, most available data come from observational studies, which are liable to confounding and reverse causation. This limitation can be overcome by the use of mendelian randomization (MR)

**What did the researchers do and find?:**

- In this MR study, the authors explored the association between higher genetically predicted BMI, fat mass, fat free mass, weight, height, and waist:hip ratio and ECG measures of atrial and ventricular conduction.
- The authors found that higher genetically predicted BMI was associated with many ECG indices that have been linked to arrhythmic risk and that this was driven by fat mass, fat free mass, and height.

**What do the findings mean?:**

- In this study, lean body mass traits appear to correlate with proarrhythmic electrophysiological remodeling.
- These data may help to risk stratify individuals who would benefit from targeted weight management interventions.
- The main limitations are inclusion of data from only predominantly European ancestry populations, and the inability to establish whether a single body size measure might be the most important in driving the associations.

## Introduction

Obesity is a growing global health problem associated with high rates of cardiometabolic morbidity [1–3] and is an independent risk factor for atrial [4] and ventricular arrhythmias [5]. Previous observational studies have identified an association between increasing adiposity and abnormalities on the 12-lead electrocardiogram (ECG) [6], such as longer P-wave duration (PWD) and increased P-wave dispersion (signifying delayed atrial conduction) [7,8] and increased QT dispersion and prolongation of corrected QT interval (QTc) (markers of abnormal ventricular activation and repolarization) [9–11]. These ECG changes are of clinical importance, as prior mendelian randomization (MR) studies have demonstrated their causal relevance on multiple adverse outcomes such as atrial fibrillation (AF) [12] and sudden cardiac death (SCD) [13].

However, body mass index (BMI) is not only determined by adiposity. Additional anthropometric measures, such as fat mass, fat-free mass, and height, are important determinants of BMI. Although it is thought that the association between BMI and electrophysiological remodeling is strongly mediated by adipose tissue, the other determinants of BMI above may also play an important role, and the potential influence of these parameters on ECG indices remains relatively less explored. Additionally, beyond overall adiposity, its distribution might play an important role in determining arrhythmic risk. This is clinically relevant given that visceral fat is thought to be more detrimental to health than subcutaneous adiposity [14]. From the current well-established association of BMI with proarrhythmic ECG changes, it is therefore impossible to establish whether it is truly only increasing adiposity that influences electrophysiological remodeling, and beyond this, it is difficult to establish whether this association is truly causal and independent of other concurrent cardiometabolic risk factors. For example, higher BMI is associated with multiple morbidities such as hypertension and ischemic heart disease, which also induce adverse cardiac remodeling [3]. In turn, these associations may also be at least partly mediated by an intermediate risk factor, such as type 2 diabetes. In the setting of observational data, it is difficult to accurately characterize these complex causal relationships.

MR is a genetic epidemiological method that can provide estimates of the effects of risk factors on an outcome using a framework that is less liable to influence by reverse causation and confounding [15]. MR leverages the random process of allele assortment and conception, which leads to an effective “randomization” of individuals to high or low genetic risk of a risk factor. This genetic liability can then be used as a proxy for the exposure itself, in an instrumental variable analysis framework. Since the initial genetic risk allocation is almost entirely random, similar to randomization in a clinical trial, it is not influenced by confounding or reverse causation. Thus, the MR framework can be used to investigate the causal relevance of the exposure on an outcome under a set of key assumptions.

The aim of this study was to utilize MR to explore the association of anthropometric metrics and on PWD, PR interval, QRS duration, and QTc interval, to elucidate the effect of body size and composition on proarrhythmic electrophysiological remodeling. Uniquely, in addition to the commonly used metric of BMI, we sought to investigate the specific effects of its determinants of height, fat mass, and fat-free mass and to explore the influence of adiposity distribution through exploring BMI-adjusted waist:hip ratio (aWHR). At present, despite the extensive observational evidence linking higher BMI to changes in ECG measures, no study, observational or MR based, has explored this.

## Methods

### Ethics and data access

This study used publicly available genome-wide association summary data. All included studies had gained ethical approval and participant consent according to individual protocols available at the referenced publications. The study is reported on the basis of the Strengthening the Reporting of Observational Studies in Epidemiology using Mendelian Randomization (STROBE-MR) Guidelines (S1 STROBE Checklist) [16]. All analyses were carried out on R version 4.1.2 [17] using the TwoSampleMR [18] and Mendelianrandomization packages [19]. No protocol was preregistered.

### Instrumental variable selection

Genome-wide significant (*p* < 5 × 10^−8^) instrumental variables for BMI (in kg/m^2^) [20], aWHR (in cm:cm) [20], and height (in inverse normal transformed and standardized cm) [21] were extracted from summary statistics of published genome-wide association studies (GWAS), respectively, including 806,834 participants, 697,734 participants, and 709,594 participants. Genome-wide significant (*p* < 5 × 10^−8^) instrumental variables for weight (in inverse normal rank transformed kg, *n* = 360,116), whole-body fat mass (in inverse normal rank transformed kg, *n* = 354,244), and whole-body fat-free mass (in inverse normal rank transformed kg, *n* = 354,808) were extracted from Neale lab UK Biobank GWAS summary statistics (http://www.nealelab.is/uk-biobank/). Whole-body fat mass and fat-free mass were measured using impedance, which were measured as outlined in the protocol and original paper [22].

Genetic association estimates for the outcomes were extracted from GWAS of outcomes, including PR interval and QRS duration (ms, *n* = 180,574 participants) [23], PWD (*n* = 44,456 participants) [24], and QTc (*n* = 84,630) [25]. The ECG measures in the original GWAS studies were derived from 12-lead ECG at rest taken in the supine position. Extraction of ECG measures, such as P-wave indices, was performed using a variety of software algorithms that are described in detail in the individual GWAS papers [23–25].

All GWAS studies for both exposures and outcomes were performed within the scope of large, multicenter population-based studies. Further details on study cohorts are available at the respective publications and are provided in Table 1.

**Table 1:** Information on the studies and consortia from which genetic association data were obtained.

Phenotype	Study	Ancestry	Units	Participants	Key features	Setting	Pubmed ID/Link
**Exposures**							
**BMI**	Pulit et al, 2018 [20]	EUR	Standardized kg/m^2^	806,834	UKB: 40–69 year population sample GIANT international consortium: multiple contributing studies	UKB: Population based, recruitment 2006–2010 GIANT international consortium, Varied age, ethnicity and sex criteria	30239722
**Waist:hip ratio**			Ratio unit, cm:cm	697,734			
**Height**	Yengo et al, 2018 [21]		Inverse normal transformed, standardized cm	709,594			30124842
**Fat mass**	Neale lab, 2021 (http://www.nealelab.is/uk-biobank/)	EUR	Inverse normal rank transformed kg	354,244	UKB: 40–69 year population sample	UKB: Population based, recruitment 2006–2010	http://www.nealelab.is/uk-biobank/
**Fat-free mass**				354,808			
**Weight**				360,116			
**Outcomes**							
**P wave duration**	Christophersen et al, 2018 [24]	EUR	Milliseconds (ms)	44,456	12 cohorts: ARIC, CHS, ERF, FHS, KORA, GHS I, MESA, Rotterdam Studies I, II, III, SHIP, GARNET, MOPMAP, SHARE	Population based Varied age, ethnicity, and sex criteria	28794112
**PR interval**	Wojcik et al, 2019 [23]	Multi-ethnic	Milliseconds (ms)	180,574	WHI, MEC, HCHS/SOL, BioMe	Population based Varied age, ethnicity and sex criteria	31217584
**QRS duration**							
**QTc interval**	Nauffal et al, 2022 [25]	EUR	Milliseconds (ms)	84,630	UKB: 40–69 year population sample	UKB: Population based, recruitment 2006–2010	35389749

ARIC, Atherosclerosis Risk in Communities; BioMe, Icahn School of Medicine at Mount Sinai BioMe biobank in New York City; CHS, Cardiovascular Health Study; ERF, Erasmus Rucphen Family; EUR, European; FHS, Framingham Heart Study; GARNET, Genome-wide Association Research Network; GHS I, Gutenberg Health Study I; GIANT, Genetic Investigation of ANthropometric Traits (https://portals.broadinstitute.org/collaboration/giant/index.php/GIANT_consortium); HCHS/SOL, Hispanic Community Health Study/Study of Latinos; KORA, Cooperative Health Research in the Augsburg Region; MEC, Multiethnic Cohort; MESA, Multi-Ethnic Study of Atherosclerosis; MOPMAP, Modification of Particulate Matter-Mediated Arrhythmogenesis in Populations; ms, milliseconds; SHARE, SNP Health Association Resource Project; SHIP, Study of Health in Pomerania; UKB, UK Biobank; WHI, Women’s Health Initiative.

### Harmonization and clumping

Gene-exposure association estimates for each exposure were harmonized with gene-outcome association estimates for corresponding instrumental SNPs in the outcome data. An attempt was made to infer positive strand alleles during harmonization. Where this could not be inferred, or if SNPs were palindromic or ambiguous, the SNP was excluded. Only SNPs with available gene-exposure and gene-outcome association estimates were included; if there were no matching SNPs for an instrumental variable in the outcome data, proxies were not sought. After harmonization, SNPs were clumped to retain only uncorrelated variants (pairwise linkage disequilibrium r^2^ < 0.001). Instrument strength was quantified using F-statistics.

### Statistical analysis

Inverse-variance weighted (IVW) MR with multiplicative random effects [26] was used as the primary analysis method for all models, to estimate the association between each genetically predicted anthropometric trait and ECG phenotype [15]. Results are presented as beta coefficients (β) with respective 95% confidence intervals (95%CI), which can be interpreted as the expected change in ECG interval, in ms, per 1-unit change in the exposure. The units for each exposure are reported in Table 1.

There are 3 core assumptions of the IVW MR approach that, if not met, can lead to unreliable results. These include the following:

1. That instrumental variables predict the exposure;
2. That instrumental variables are not associated with confounders of the association between the exposure and outcome; and
3. That instrumental variables are only associated with the outcome through the exposure.

The first assumption was tested by quantification of instrument strength using F-statistics. In instances where instrumental SNPs influence the outcome through additional biological pathways that are parallel to, but do not act through the exposure, these assumptions are violated in a phenomenon called horizontal pleiotropy. Sensitivity analysis using weighted median MR [27] and MR-Egger can be used to explore this phenomenon. The weighted median method has been shown to provide consistent estimates even in situations where up to half of the instrumental SNPs are invalid or horizontally pleiotropic [27]. Additionally, the MR-Egger method can be used to more formally test for the presence of directional pleiotropy through the addition and testing of an intercept term, though the method relies on the weaker assumption that the instrument strength is independent of direct effects (InSIDE assumption) [28].

Statistical significance was considered at an alpha value of 0.0021 after Bonferroni adjustment for testing of 24 hypotheses (6 exposures on 4 outcomes, 0.05/24).

## Results

### P-wave duration

**Fig 1:**
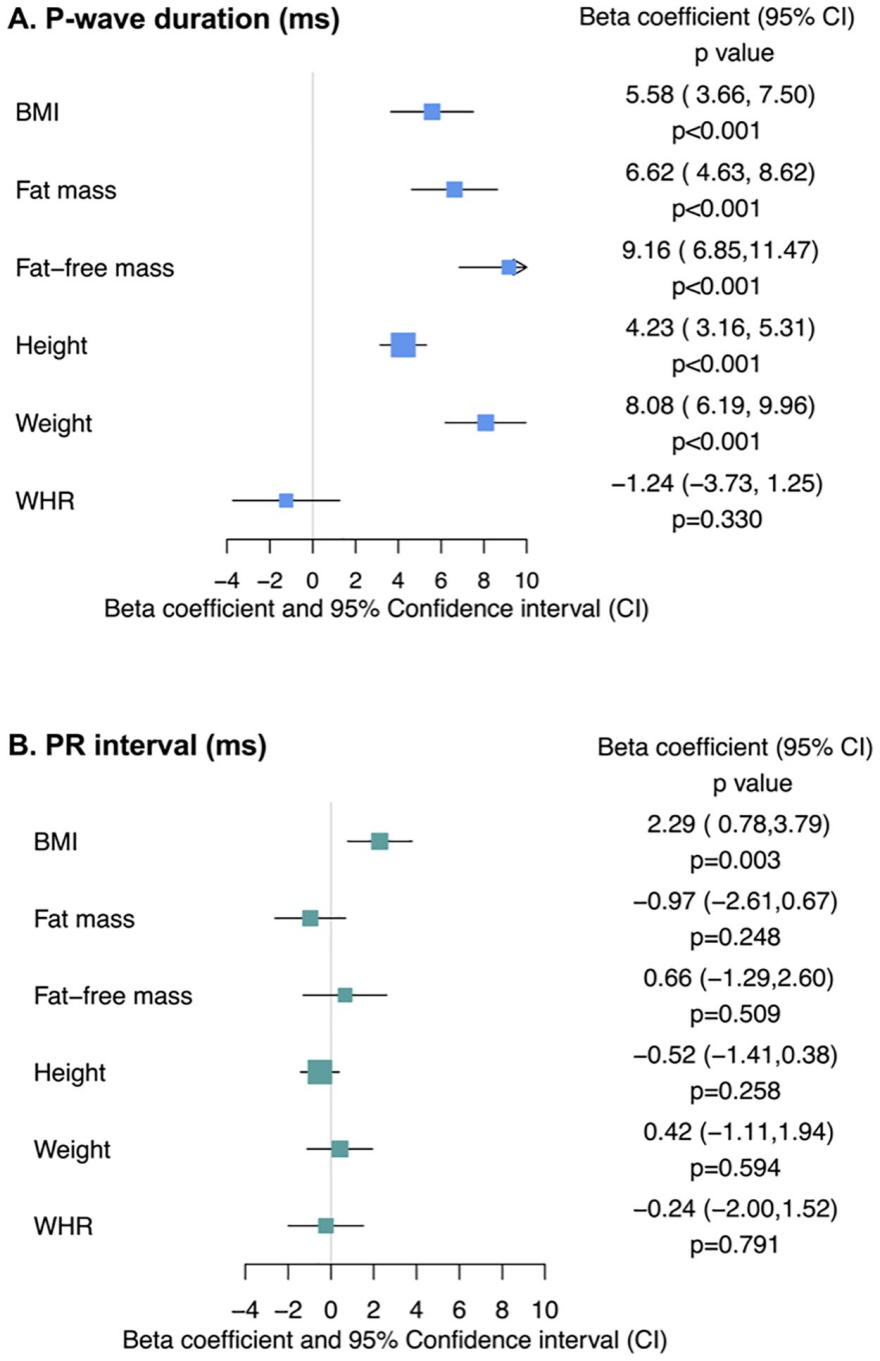
MR estimates for the effects of genetically predicted BMI on PWD and PR interval. aWHR, adjusted waist:hip ratio; BMI, body mass index; CI, confidence interval; MR, mendelian randomization; ms, milliseconds; PWD, P-wave duration.

Higher genetically predicted BMI was associated with longer PWD (β 5.58; 95%CI [3.66,7.50]; *p* < 0.001), as was higher genetically predicted fat mass (β 6.62; 95%CI [4.63,8.62]; *p* < 0.001) and fat-free mass (β 9.16; 95%CI [6.85,11.47]; *p* < 0.001). Genetically predicted height (β 4.23; 95%CI [3.16, 5.31]; *p* < 0.001) and weight (β 8.08; 95%CI [6.19,9.96]; *p* < 0.001) were also associated with longer PWD. However, genetically predicted aWHR was not associated with differences in PWD (β −1.24; 95%CI [−3.73,1.25]; *p* = 0.330). The results are displayed in Fig 1 and Table 2.

**Table 2:** Univariable MR estimates for the effects of genetically predicted anthropometric traits on ECG parameters, using an IVW model with multiplicative random effects.

Exposure	Outcome	nSNP	β	95% CI	*P* value
**BMI (1-unit, standardized kg/m** ^**2**^ **)**	PWD	202	5.58	3.66, 7.50	1.23 × 10^−8^
	PR interval	512	2.29	0.78, 3.79	0.003
	QRS duration	424	0.06	−0.62, 0.74	0.856
	QT interval	507	3.53	2.63, 4.43	1.27 × 10^−14^
**Whole-body fat mass (1-unit inverse normal rank transformed kg)**	PWD	136	6.62	4.63, 8.62	8.17 × 10–11
	PR interval	323	−0.97	−2.61, 0.67	0.248
	QRS duration	201	−0.20	−0.96, 0.55	0.599
	QT interval	318	3.65	2.73, 4.57	8.42 × 10^−15^
**Whole-body fat-free mass (1-unit inverse normal rank transformed kg)**	PWD	203	9.16	6.85, 1.47	8.13 × 10^−15^
	PR interval	468	0.66	−1.29, 2.60	0.509
	QRS duration	135	−0.01	−1.35, 1.33	0.989
	QT interval	468	2.08	0.85, 3.31	0.001
**Height (1-unit inverse normal transformed, standardized height, cm)**	PWD	418	4.23	3.16, 5.31	1.26 × 10–14
	PR interval	837	−0.52	−1.41, 0.38	0.258
	QRS duration	137	−0.59	−1.52, 0.34	0.211
	QT interval	832	0.98	0.46, 1.50	2.39 × 10^−4^
**Weight (1-unit, inverse normal rank transformed kg)**	PWD	159	8.08	6.19, 9.96	4.55 × 10^−17^
	PR interval	374	0.42	−1.11, 1.94	0.594
	QRS duration	204	−0.24	−1.08, 0.59	0.567
	QT interval	371	3.45	2.54, 4.36	9.58 × 10^−14^
**aWHR (1-unit, wcm:hcm)**	PWD	124	−1.24	−3.73, 1.25	0.330
	PR interval	305	−0.24	−2.00, 1.52	0.791
	QRS duration	46	0.69	−1.09, 2.48	0.445
	QT interval	302	1.92	0.87, 2.97	3.38 × 10^−4^

aWHR, adjusted waist:hip ratio; BMI, body mass index; β, Beta coefficient (ms per 1-unit exposure increase); CI, confidence interval; ECG, electrocardiogram; IVW, inverse-variance weighted; MR, mendelian randomization; nSNP, number of instrumental SNPs; PWD, P-wave duration.

As displayed in Table 3, the results remained consistent on sensitivity analyses using weighted median MR. MR-Egger estimates also remained consistent, and intercept test did not identify evidence of directional pleiotropy in the association with BMI (*p* = 0.534), height (*p* = 0.107), fat mass (*p* = 0.657), fat-free mass (*p* = 0.326), weight (*p* = 0.292), and aWHR (*p* = 0.748). Instrumental variable F-statistics were >10 in all cases, as reported in Table 4.

**Table 3:** MR sensitivity analyses for effects of genetically predicted anthropometric traits on ECG parameters, using weighted median MR and MR-Egger models.

Exposure	Outcome	Method	β	Std. Error	*P* value
**Body mass index (1-unit BMI in kg/m** ^**2**^ **)**	PWD	Weighted median	10.14	0.90	1.76 × 10^−29^
		MR-Egger	6.99	2.47	0.005
				intercept	0.534
	PR interval	Weighted median	1.83	1.16	0.115
		MR-Egger	0.98	2.07	0.636
				intercept	0.498
	QRS duration	Weighted median	−0.30	0.60	0.616
		MR-Egger	0.27	0.91	0.765
				intercept	0.803
	QT interval	Weighted median	3.53	0.46	1.27 × 10^−14^
		MR-Egger	3.10	1.23	0.012
				intercept	0.709
**Fat mass (1-unit inverse normal rank transformed fat mass in kg)**	PWD	Weighted median	10.60	0.90	2.89 × 10^−32^
		MR-Egger	5.28	3.19	0.101
				intercept	0.657
	PR interval	Weighted median	−0.47	1.2	0.715
		MR-Egger	−0.70	2.64	0.792
				intercept	0.913
	QRS duration	Weighted median	−0.39	0.62	0.526
		MR-Egger	−0.90	1.19	0.451
				intercept	0.537
	QT interval	Weighted median	3.65	0.47	8.42 × 10^−15^
		MR-Egger	1.48	1.41	0.297
				intercept	0.104
**Fat-free mass (1-unit inverse normal rank transformed fat-free mass in kg)**	PWD	Weighted median	13.52	1.00	3.09 × 10^−41^
		MR-Egger	11.94	3.06	1.29 × 10^−4^
				intercept	0.326
	PR interval	Weighted median	1.76	1.39	0.207
		MR-Egger	−0.49	2.58	0.851
				intercept	0.631
	QRS duration	Weighted median	−0.13	1.01	0.894
		MR-Egger	−0.16	1.78	0.929
				intercept	0.927
	QT interval	Weighted median	2.08	0.63	9.52 × 10^−4^
		MR-Egger	1.97	1.59	0.215
				intercept	0.939
**Height (1-unit inverse normal transformed, standardized height in cm)**	PWD	Weighted median	6.03	0.49	7.80 × 10^−35^
		Mr-Egger	2.64	1.13	0.020
				intercept	0.107
	PR interval	Weighted median	−0.92	0.71	0.193
		Mr-Egger	0.13	0.97	0.893
				intercept	0.451
	QRS duration	Weighted median	−0.31	0.70	0.659
		Mr-Egger	0.41	1.01	0.683
				intercept	0.263
	QT interval	Weighted median	0.98	0.27	2.39 × 10^−4^
		Mr-Egger	1.84	0.57	0.001
				intercept	0.088
**Weight (1-unit inverse normal rank transformed weight in kg)**	PWD	Weighted median	11.15	0.86	1.23 × 10^−38^
		Mr-Egger	5.47	2.65	0.040
				intercept	0.292
	PR interval	Weighted median	0.88	1.25	0.481
		Mr-Egger	−0.41	2.21	0.852
				intercept	0.689
	QRS duration	Weighted median	−0.16	0.67	0.813
		Mr-Egger	−0.26	1.19	0.827
				intercept	0.987
	QT interval	Weighted median	3.45	0.46	9.58x10^−14^
		Mr-Egger	2.26	1.25	0.072
				intercept	0.305
**aWHR (1-unit increase, wcm:hcm)**	PWD	Weighted median	−7.40	1.35	4.11 × 10^−8^
		Mr-Egger	−2.25	3.39	0.508
				intercept	0.748
	PR interval	Weighted median	0.66	1.33	0.620
		Mr-Egger	3.54	2.31	0.127
				intercept	0.078
	QRS duration	Weighted median	1.94	1.16	0.096
		Mr-Egger	4.22	2.13	0.054
				intercept	0.076
	QT interval	Weighted median	1.92	0.54	3.38 × 10^−4^
		Mr-Egger	2.58	1.29	0.047
				intercept	0.575

aWHR, adjusted waist:hip ratio; BMI, body mass index; β, Beta coefficient; ECG, electrocardiogram; MR, mendelian randomization; PWD, P-wave duration; Std. Error, standard error.

**Table 4:** Instrument strength for study exposures.

Phenotype	Participants	IV F-statistic	Proportion of variance explained by IVs (R^2^, %)
**Exposures**			
BMI	806,834	78.32	5.07
Height	709,594	224.76	21.08
Fat mass	354,244	58.67	5.08
Fat-free mass	354,808	81.21	9.69
Weight	360,116	65.84	6.45
aWHR	697,734	89.47	4.08

aWHR, adjusted waist:hip ratio; BMI, body mass index; IV, instrumental variable.

### PR interval

Higher genetically predicted BMI was associated with longer PR interval (β 2.29; 95%CI [0.78,3.79]; *p* = 0.003), but this was no longer significant after accounting for multiple testing (threshold *p*-value = 0.0021). No association was observed between higher genetically predicted fat mass (β −0.97; 95%CI [−2.61,0.67]; *p* = 0.248), fat-free mass (β 0.66; 95%CI [−1.29,2.60]; *p* = 0.509), height (β −0.52; 95%CI [−1.41,0.38]; *p* = 0.258), weight (β 0.42; 95%CI [−1.11,1.94]; *p* = 0.594), or aWHR (β −0.24; 95%CI [−2.00,1.52]; *p* = 0.791) and PR interval. The results are displayed in Fig 1 and Table 2.

As displayed in Table 3, the results remained consistent on sensitivity analyses using weighted median MR and MR-Egger. MR-Egger intercept test did not identify evidence of directional pleiotropy in the association with BMI (*p* = 0.498), height (*p* = 0.451), fat mass (*p* = 0.631), fat-free mass (*p* = 0.913), weight (*p* = 0.689), and aWHR (*p* = 0.078). F-statistics for all instruments were >10, as reported in Table 4.

### QRS duration

**Fig 2:**
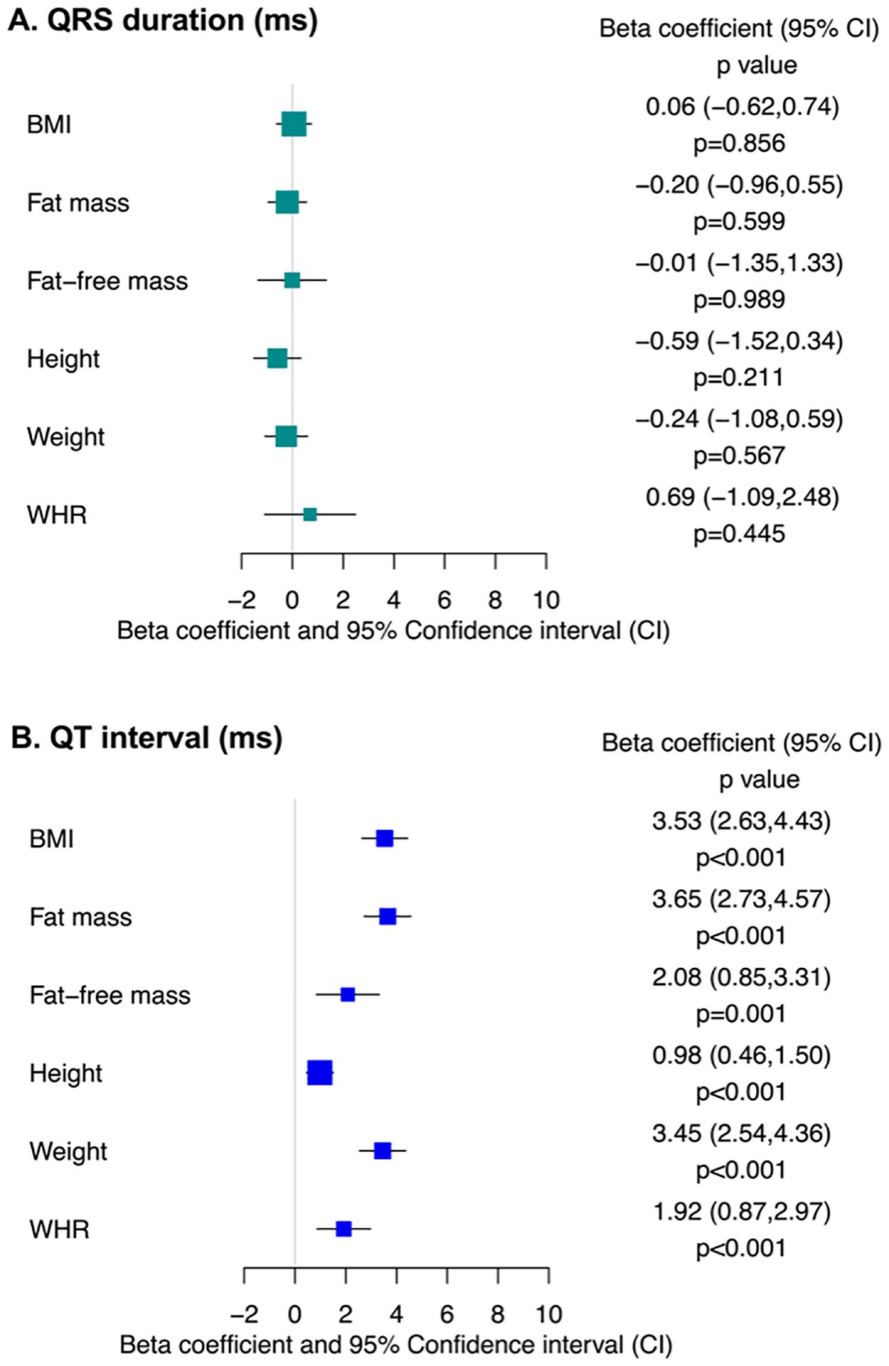
MR estimates for the effects of genetically predicted BMI on QRS duration and QT interval. aWHR, adjusted waist:hip ratio; BMI, body mass index; CI, confidence interval; MR, mendelian randomization; ms, milliseconds.

Higher genetically predicted BMI was not associated differences in QRS interval (β 0.06; 95%CI [−0.62,0.74]; *p* = 0.856) and neither were genetically predicted fat mass (β −0.20; 95%CI [−0.96,0.55]; *p* = 0.599), fat-free mass (β −0.01; 95%CI [−1.35,1.33]; *p* = 0.989), height (β −0.59; 95%CI [−1.52,0.34]; *p* = 0.211), weight (β −0.24; 95%CI [−1.08,0.59]; *p* = 0.567), and aWHR (β 0.69; 95%CI [−1.09,2.48]; *p* = 0.445). The results are displayed in Fig 2 and Table 2.

As displayed in Table 3, the results remained consistent on sensitivity analyses using weighted median MR. MR-Egger estimates also remained consistent, and intercept test did not identify evidence of directional pleiotropy in the association with BMI (*p* = 0.802), height (*p* = 0.263), fat mass (*p* = 0.537), fat-free mass (*p* = 0.927), weight (*p* = 0.987), and aWHR (*p* = 0.076). F-statistics for all instruments were >10, as reported in Table 4.

### Corrected QT interval

Higher genetically predicted BMI was associated with longer QTc (β 3.53; 95%CI [2.63,4.43]; *p* < 0.001), driven by both genetically predicted fat mass (β 3.65; 95%CI [2.73,4.57]; *p* < 0.001) and fat-free mass (β 2.08; 95%CI [0.85,3.31]; *p* = 0.001). Additionally, genetically predicted height (β 0.98; 95%CI [0.46,1.50]; *p* < 0.001), weight (β 3.45; 95%CI [2.54,4.36]; *p* < 0.001), and aWHR (β 1.92; 95%CI [0.87,2.97]; *p* < 0.001) were all associated with longer QTc. The results are displayed in Fig 2 and Table 2.

As displayed in Table 3, the results remained consistent on sensitivity analyses using weighted median MR. MR-Egger estimates also remained consistent, and intercept test did not identify evidence of directional pleiotropy in the association with BMI (*p* = 0.709), height (*p* = 0.088), fat mass (*p* = 0.704); fat-free mass (*p* = 0.939), weight (*p* = 0.305), and aWHR (*p* = 0.575). F-statistics for all instruments were >10, as reported in Table 4.

## Discussion

In this study, we used MR to investigate the causal relevance of multiple anthropometric traits, relating to adiposity and lean body composition, and 12-lead ECG indices that are associated with atrial and ventricular arrhythmias. The main findings may be summarized in 4 key points. First, the results support a causal nature of the association between adiposity and prolonged PWD but do not support a causal role of adiposity on PR interval. Second, they reveal an association between higher lean mass and height with PWD but not PR interval. Third, higher adiposity was associated with a prolonged QTc interval but was not associated with a longer QRS interval. Finally, we demonstrate an association between lean mass and height with prolonged QTc, which has not been previously reported.

The causal inferences that can be made on the basis of MR study results is reliant on meeting a number of instrumental variant assumptions. These state that the instruments must predict the exposure, that they must be associated with the outcome only through the exposure, and that no confounders or common causes of the exposure, in this case the genetic variants, and the outcome exist. In order to address the first assumption, we formally quantified the F-statistics and detected no evidence of weak instruments that might bias the results. To evaluate the second assumption, sensitivity analyses were carried out. These only identified evidence of horizontal pleiotropy for the association of BMI with PR interval, suggesting that this association might be biased, and, therefore, this result should not be taken to support causal relevance. Importantly, however, it is known that MR-Egger performs less well in settings where there is significant overlap between the GWAS studies used to source gene-exposure and gene-outcome association estimates. Since this is the case in the present study, it cannot be excluded that some pleiotropy might exist for other associations [29]. To further investigate, we also performed weighted median MR analyses to corroborate findings from MR-Egger, and this provides further reassurance that directional pleiotropy is unlikely to have occurred undetected. The final assumption cannot formally be tested but can be mitigated by the use of GWAS data from similar ancestries. For this reason, we only utilized data from studies in European ancestry individuals. However, for some data sources, specifically that of PR interval and QRS duration, there was a proportion of non-European ancestry individuals included in the study due to lack of availability of data in European ancestry individuals only. Though only a minority of participants were non-European ancestry in these studies, it should be considered that some bias might arise from this.

The results of this study support a causal nature of the association between BMI and slower atrial conduction. Prior observational studies have outlined an association between adiposity traits, including weight, BMI, and fat mass, on atrial depolarization, slower atrial conduction, most commonly describing longer PWD [8,11,30–33] in individuals with higher BMI. The results of our study suggests that weight control is likely to play an important role in reduction of atrial arrhythmic risk in individuals with obesity and optimization of rhythm control strategies in AF. Promising evidence has established a degree of reversibility of these atrial ECG changes following weight loss [8,34–37]. Combined with prior observational evidence linking longer PWD with greater AF risk and higher recurrence rates after catheter ablation [38–46], our results provide further evidence to support the central role of weight reduction to reduce both the risk of onset and recurrence of atrial arrhythmias.

Importantly, the results suggest that adiposity traits predominantly associate with PWD, with no evidence association with PR interval. We therefore suggest that the potential influence of anthropometric traits is likely predominantly though electrophysiological and structural remodeling of the atria (e.g., fibrosis, dilatation) [47], resulting in slower atrial conduction, rather than through direct influence on the specialized conduction tissue, as the AV node is the major determinant of PR interval. Mechanistically, epicardial fat might play a key role, as it is known to influence cardiac electrophysiology by modulating ionic currents via paracrine mechanisms [6]. Importantly, as previously noted, the GWAS for PR interval was in a multi-ancestry cohort, so the null findings should not be interpreted as conclusive evidence of lack of association and should be replicated in a European ancestry cohort when this becomes available.

In contrast to the results of our study, a prior MR investigation identified an inverse, rather than direct, association between PWD, PR interval, and AF risk [12]. This is not intuitively in agreement with our results. Higher BMI has a well-established causal effect on AF. In this study, we also demonstrate that higher BMI causes slower atrial depolarization, with higher PWD and PR interval. Thus, we would expect that higher, rather than lower, PWD and PR interval might be associated with higher risk of AF. This discrepancy is most likely explained by the existence of a nonlinear association between atrial ECG indices and AF risk, whereby both individuals at the low and high end of the distribution are at increased risk of AF. However, given the current lack of evidence to demonstrate this, it is an important target for further research as duly highlighted by the authors in the original study.

Together with the findings on adiposity-related traits, our results support an additional role of both fat-free mass and height on increased PWD. Height and fat-free body mass are 2 closely correlated phenotypes [48]. Although the association between these anthropometric traits and ECG indices of atrial conduction has not been described previously, studies have reported a stronger contribution of lean body mass on excess AF risk conferred by BMI [49–52] compared to fat mass. Similarly, height has been associated with greater risk of AF in observational and MR studies [53–61]. Although the mechanisms underlying these associations are unclear, a possible explanation relates to left atrial volume. Indeed, the association between left atrial volumes and AF risk is well established [62]. Taller individuals have higher absolute left atrial volumes since cardiac chamber volume is a direct function of body size [63]. It is possible that, despite the nonpathological nature of the increased volume conferred by height, it nevertheless contributes to a degree of electrophysiological dysfunction and arrhythmia predisposition [63], as suggested by the association with longer PWD described in the MR analyses this study. Overall, the mechanism behind this association remains an important unanswered question and a key target for further exploration.

There were no associations between any of the anthropomorphic traits studied and QRS duration. Since ventricular depolarization is facilitated by the His–Purkinje network, this suggests that the effects of body composition are more pronounced on ventricular myocardium than on specialized conduction tissue. The observational evidence of associations between adiposity and QRS duration is mixed, with some studies finding no association [64] and another reporting an association between increasing BMI and QRS duration that was independent of other covariates such as sex and age [65]. Our study adds to this contrasting evidence by suggesting a lack of causal association between adiposity and QRS duration. However, it must be noted that the GWAS study for QRS interval was performed a multi-ancestry cohort; therefore, the null findings should not be interpreted as conclusive evidence of lack of association and should be replicated in a European ancestry cohort when this becomes available.

Several observational studies have described direct associations between BMI and QTc [9,11,66], with a degree of reversibility after significant weight loss [66]. Another recent observational study using UK Biobank data also showed that QTc interval prolongs with increasing BMI, body fat, waist:hip ratio, as well as hip and waist circumference [67]. The association between longer QTc and risk of SCD is well established and supported by MR evidence [13]. In line with this, obesity has been associated with a higher risk of ventricular arrhythmias in prior observational studies [68] and is the most common ischemic cause of SCD [69] even after adjusting for age, sex, ethnicity, and cardiovascular risk factors [14]. The results of the present study support that this previously described association might be of causal relevance. The results specifically demonstrate an association between BMI and QTc, and additionally of both total fat mass and aWHR with QTc, suggesting an important role of both the volume of adiposity and its distribution. Considering the established association between QTc and SCD, as well as the reversibility in ECG phenotypes demonstrated after significant weight loss, the results herein stress the importance of weight reduction on reducing risk of SCD in individuals with obesity. Though SCD remains a rare cause of mortality, targeted weight loss intervention in those at high-risk, such as those with prior myocardial infarction or reduced ejection fraction, is likely of important benefit and should be a key priority for randomized study.

The association of lean body mass and height with ventricular repolarization is less well described than that with adiposity. To date, there are no published studies, observational or MR based, assessing the association of height and lean body mass with ventricular ECG parameters. However, observational evidence exists describing of greater burden of ventricular ectopics with increasing height [70,71]. Conversely, one prior study has described an inverse association between height and SCD [72]. Pathophysiologically, lean body mass and height, which are closely correlated, are known to be major predictors of absolute left ventricular mass [73]. Left ventricular mass and its diagnostic correlate of left ventricular hypertrophy are, in turn, known to be associated with longer QTc interval [74–77] and are predictors of SCD, including among young, healthy individuals and athletes [78–81]. Mechanistically, the associations between height and fat-free mass with longer QTc observed in this study may be mediated by what would be described as a “physiological” increase in left ventricular mass. Overall, the results raise the possibility that taller individuals with a greater lean body mass have a more proarrhythmic electroanatomic ventricular architecture.

There are a number of limitations to this study. First, the effect estimates presented cannot be used to infer the predicted change in ECG parameters per unit increase or decrement in the exposure phenotypes with the exceptions of BMI and aWHR. This is because the inverse normal rank transformation carried out on the exposure measures for analysis in the original GWAS studies mean that the unit changes in the exposure cannot be intuitively interpreted. Second, the outcome data sources for PR interval and QRS duration were from a GWAS on a population of mixed ancestry, while exposure data sets were on populations of European ancestry. Though both exposure and outcome estimates were adjusted for population structure, there can be residual bias in the MR estimates from population stratification. This can act in either direction (both away from the null or toward the null), and, therefore, the null results on the analyses on these outcomes should not be taken as unequivocal evidence of lack of an association. Third, we attempted to further explore the mechanistic role of left atrial size and ventricular mass in the associations demonstrated in this study, but there were insufficient instruments to carry out MR analysis in the largest available GWAS to date [82]. Fourth, we attempted to perform multivariable analyses including multiple anthropometric traits that are known to be correlated (e.g., fat mass and fat-free mass) in an attempt to establish the key driving factors in the associations with PWD and QTc. Unfortunately, the majority of these analyses had weak instruments (conditional F-statistics <10) and therefore were unreliable. The results of this analysis are reported in S1 Table This remains an important target for future research once larger GWAS data becomes available. Fifth, the use of predominantly European ancestry specific data limits generalisability to other ancestries; the analysis should therefore be replicated using data from other ancestries once available. Sixth, in this study, there was a degree of sample overlap, as both the exposure and outcome data sets included UK Biobank participants. The potential bias that might stem from this is, however, limited, as sample overlap has been shown to exert a very limited influence on results in the setting of large biobanks even when complete sample overlap exists [29]. Finally, the GWAS studies that were used to derive gene-outcome association data utilized a range of methods and software programs to extract the ECG indices. Within the scope of our study, we did not have access to individual-level data and therefore could not access the ECG tracings, and for this reason, we were unable to recalculate the ECG indices using different techniques such as signal averaging, which is known to be particularly useful in individuals with obesity.

Our results support causal associations between multiple anthropometric traits, both adiposity- and non-adiposity related, and abnormal ECG indices that have been previously causally related to risk of atrial and ventricular arrhythmias. The results stress the mechanistic importance of weight management in at-risk individuals to prevent or reverse proarrhythmic electrophysiological remodeling. The majority of associations related to ECG indices reflect electrophysiological function of cardiomyocytes rather than function of specialized conducting tissue. The results additionally identify an important role of fat-free mass and height, which have not been previously described as proarrhythmic factors except in the setting of AF risk. These are key targets for further investigation, as they might provide important biological insight and aid clinical risk stratification in high-risk individuals.

## Supporting information

S1 STROBE Checklist Strengthening the Reporting of Observational Studies in Epidemiology using Mendelian Randomization (STROBE-MR) checklist of recommended items to address in mendelian randomization (MR) studies. (DOCX)

S1 Table Results of multivariable mendelian randomization (MVMR) analyses. (DOCX)

## Abbreviations

AF: atrial fibrillation
aWHR: adjusted waist:hip ratio
BMI: body mass index
ECG: electrocardiogram
GWAS: genome-wide association study
IVW: inverse-variance weighted
MR: mendelian randomization
PWD: P-wave duration
QTc: corrected QT
SCD: sudden cardiac death
SNP: single nucleotide polymorphism
95%CI: 95% confidence interval
