# Supplementary material for: Multiple anthropometric measures and proarrhythmic 12-lead ECG indices: A mendelian randomization study
Source: PLoS Med. 2023 Aug 8;20(8):e1004275. doi: 10.1371/journal.pmed.1004275 (PMC10443852; doi:10.1371/journal.pmed.1004275)
Supplement: S1 STROBE Checklist — (DOCX) [file pmed.1004275.s001.docx]

**S1 STROBE Checklist - STROBE-MR checklist of recommended items to address in reports of Mendelian randomization studies**^1^ ^2^

| **Item No.** | **Section** | **Checklist item** | **Page No.** | **Relevant text from manuscript** |
| --- | --- | --- | --- | --- |
| 1 | **TITLE and ABSTRACT** | Indicate Mendelian randomization (MR) as the study’s design in the title and/or the abstract if that is a main purpose of the study | Title | Causal relevance of adiposity, lean body mass and height on proarrhythmic 12-lead ECG indices: a Mendelian randomization study |
|  | **INTRODUCTION** |  |  |  |
| 2 | **Background** | Explain the scientific background and rationale for the reported study. What is the exposure? Is a potential causal relationship between exposure and outcome plausible? Justify why MR is a helpful method to address the study question | Background Paragraph 5 | ‘The aim of this study was to explore any causal[…’ |
| 3 | **Objectives** | State specific objectives clearly, including pre-specified causal hypotheses (if any). State that MR is a method that, under specific assumptions, intends to estimate causal effects | Background Paragraph 5 | ‘The aim of this study was to explore any causal[…’ |
|  | **METHODS** |  |  |  |
| 4 | **Study design and data sources** | Present key elements of the study design early in the article. Consider including a table listing sources of data for all phases of the study. For each data source contributing to the analysis, describe the following: |  |  |
|  | a) | Setting: Describe the study design and the underlying population, if possible. Describe the setting, locations, and relevant dates, including periods of recruitment, exposure, follow-up, and data collection, when available. | Table 1 | Table 1 |
|  | b) | Participants: Give the eligibility criteria, and the sources and methods of selection of participants. Report the sample size, and whether any power or sample size calculations were carried out prior to the main analysis | Table 1 | Table 1 |
|  | c) | Describe measurement, quality control and selection of genetic variants | Methods  Instrumental variable selection  Paragraph 1 | “Genome-wide significant (p<5x10-8) instrumental variables[…]” |
|  | d) | For each exposure, outcome, and other relevant variables, describe methods of assessment and diagnostic criteria for diseases | Methods  Instrumental variable selection  Paragraph 2 | ‘Further details on study cohorts are available at the respective publications, and are provided in Table 1.’ |
|  | e) | Provide details of ethics committee approval and participant informed consent, if relevant | Methods  Ethics and data access | All included studies had gained ethical approval and participant consent according to individual protocols available at the referenced publications |
| 5 | **Assumptions** | Explicitly state the three core IV assumptions for the main analysis (relevance, independence and exclusion restriction) as well assumptions for any additional or sensitivity analysis | Methods  Statistical analysis paragraph 2 | There are three core assumptions of the IVW MR approach that, if not met, can lead to unreliable results. These include:  1. That instrumental variables predict the exposure  2. That instrumental variables are not associated with confounders of the association between the exposure and outcome  3. That instrumental variables are only associated with the outcome through the exposure |
| 6 | **Statistical methods: main analysis** | Describe statistical methods and statistics used | Methods  Statistical analysis  Paragraph 1 | Inverse-variance weighted (IVW) MR with multiplicative random effects [27] was used as the primary analysis method for all models |
|  | a) | Describe how quantitative variables were handled in the analyses (i.e., scale, units, model) | Methods  Instrumental variable selection  Paragraph 1 | Eg.  variables for body mass index (BMI, in kg/m2) [21], adjusted waist:hip ratio (aWHR, in cm:cm) [21] and height (in inverse normal transformed and standardized cm) |
|  | b) | Describe how genetic variants were handled in the analyses and, if applicable, how their weights were selected | Methods  Harmonisation and clumping | Gene-exposure association estimates for each exposure were harmonized with gene-outcome association estimates for corresponding instrumental SNPs in the outcome data. An attempt was made to infer positive strand alleles during harmonization. Where this could not be inferred, or if SNPs were palindromic or ambiguous, the SNP was excluded. Only SNPs with available gene-exposure and gene-outcome association estimates were included; if there were no matching SNPs for an instrumental variable in the outcome data, proxies were not sought. After harmonization SNPs were clumped to retain only uncorrelated variants (pair-wise linkage disequilibrium r2 <0.001). Instrument strength was quantified using F-statistics. |
|  | c) | Describe the MR estimator (e.g. two-stage least squares, Wald ratio) and related statistics. Detail the included covariates and, in case of two-sample MR, whether the same covariate set was used for adjustment in the two samples | Methods  Statistical analysis  Paragraph 1 | Inverse-variance weighted (IVW) MR with multiplicative random effects [27] was used as the primary analysis method for all models, to estimate the association between each genetically-predicted anthropometric trait and ECG phenotype [16]. |
|  | d) | Explain how missing data were addressed | NA | No missing data |
|  | e) | If applicable, indicate how multiple testing was addressed | Methods  Statistical analysis  Paragraph 4 | Statistical significance was considered at an alpha value of 0.0021 after Bonferroni adjustment for testing of 24 hypotheses (six exposures on four outcomes, 0.05/24). |
| 7 | **Assessment of assumptions** | Describe any methods or prior knowledge used to assess the assumptions or justify their validity | Methods  Instrumental variable selection  Paragraph 3 | In instances where instrumental SNPs influence the outcome through additional biological pathways that are parallel to, but do not act through the exposure, these assumptions are violated in a phenomenon called horizontal pleiotropy. Sensitivity analysis using weighted median MR [28] and MR-Egger can be used to explore this phenomenon. |
| 8 | **Sensitivity analyses and additional analyses** | Describe any sensitivity analyses or additional analyses performed (e.g. comparison of effect estimates from different approaches, independent replication, bias analytic techniques, validation of instruments, simulations) | Methods  Instrumental variable selection  Paragraph 3 | As above |
| 9 | **Software and pre-registration** |  |  |  |
|  | a) | Name statistical software and package(s), including version and settings used | Methods  Ethics and data access | All analyses were carried out on R version 4.1.2 [18] using the TwoSampleMR [19] and Mendelianrandomization packages [20]. |
|  | b) | State whether the study protocol and details were pre-registered (as well as when and where) | Methods  Ethics and data access | No protocol was pre-registered. |
|  | **RESULTS** |  |  |  |
| 10 | **Descriptive data** |  |  |  |
|  | a) | Report the numbers of individuals at each stage of included studies and reasons for exclusion. Consider use of a flow diagram | NA | NA – Two-sample |
|  | b) | Report summary statistics for phenotypic exposure(s), outcome(s), and other relevant variables (e.g. means, SDs, proportions) | NA | NA – Two-sample |
|  | c) | If the data sources include meta-analyses of previous studies, provide the assessments of heterogeneity across these studies | NA | NA – no meta-analysis performed |
|  | d) | For two-sample MR:  i.  Provide justification of the similarity of the genetic variant-exposure associations between the exposure and outcome samples  ii.  Provide information on the number of individuals who overlap between the exposure and outcome studies | Discussion  Limitations paragraph | Sixth, in this study there was a degree of sample overlap, as both the exposure and outcome datasets included UK Biobank participants. The potential bias that might stem from this is, however, limited, as sample overlap has been shown to exert a very limited influence on results in the setting of large biobanks even when complete sample overlap exists [82]. |
| 11 | **Main results** |  |  |  |
|  | a) | Report the associations between genetic variant and exposure, and between genetic variant and outcome, preferably on an interpretable scale | NA | NA – publicly available, thousands of SNPs therefore unable to include as supplement |
|  | b) | Report MR estimates of the relationship between exposure and outcome, and the measures of uncertainty from the MR analysis, on an interpretable scale, such as odds ratio or relative risk per SD difference | Results | Results section |
|  | c) | If relevant, consider translating estimates of relative risk into absolute risk for a meaningful time period | NA | NA – two-sample |
|  | d) | Consider plots to visualize results (e.g. forest plot, scatterplot of associations between genetic variants and outcome versus between genetic variants and exposure) | Figures | Figures 1 and 2 |
| 12 | **Assessment of assumptions** |  |  |  |
|  | a) | Report the assessment of the validity of the assumptions | Methods/Results | The first assumption was tested by quantification of instrument strength using F-statistics. In instances where instrumental SNPs influence the outcome through additional biological pathways that are parallel to, but do not act through the exposure, these assumptions are violated in a phenomenon called horizontal pleiotropy. Sensitivity analysis using weighted median MR [28] and MR-Egger can be used to explore this phenomenon.  Table 2  Table 3 |
|  | b) | Report any additional statistics (e.g., assessments of heterogeneity across genetic variants, such as *I^2^*, Q statistic or E-value) | Tables | Table 2 |
| 13 | **Sensitivity analyses and additional analyses** |  |  |  |
|  | a) | Report any sensitivity analyses to assess the robustness of the main results to violations of the assumptions | Table 2 | Table 2 |
|  | b) | Report results from other sensitivity analyses or additional analyses | NA | NA |
|  | c) | Report any assessment of direction of causal relationship (e.g., bidirectional MR) | NA | NA – not biologically plausible |
|  | d) | When relevant, report and compare with estimates from non-MR analyses | NA | Discussion  Observational estimates only available for BMI |
|  | e) | Consider additional plots to visualize results (e.g., leave-one-out analyses) | NA | NA – to many IVs to perform LOO analysis |
|  | **DISCUSSION** |  |  |  |
| 14 | **Key results** | Summarize key results with reference to study objectives | Discussion  Paragraph 1 | In this study, we used MR to investigate the causal association between multiple anthropometric traits, relating to adiposity and lean body composition, and 12-lead ECG indices that are associated with atrial and ventricular arrhythmias. The main findings may be summarized in four key points. |
| 15 | **Limitations** | Discuss limitations of the study, taking into account the validity of the IV assumptions, other sources of potential bias, and imprecision. Discuss both direction and magnitude of any potential bias and any efforts to address them | Discussion  Paragraph 2 | The causal inferences that can be made on the basis of MR study results is reliant on meeting a number of instrumental variant assumptions.[…]. |
| 16 | **Interpretation** |  |  |  |
|  | a) | Meaning: Give a cautious overall interpretation of results in the context of their limitations and in comparison with other studies | Discussion  Paragraph 2,3,6,7,8 | See Discussion |
|  | b) | Mechanism: Discuss underlying biological mechanisms that could drive a potential causal relationship between the investigated exposure and the outcome, and whether the gene-environment equivalence assumption is reasonable. Use causal language carefully, clarifying that IV estimates may provide causal effects only under certain assumptions | Discussion  Paragraph 4,5,9 | See Discussion |
|  | c) | Clinical relevance: Discuss whether the results have clinical or public policy relevance, and to what extent they inform effect sizes of possible interventions | Discussion  Final paragraph | See Discussion |
| 17 | **Generalizability** | Discuss the generalizability of the study results (a) to other populations, (b) across other exposure periods/timings, and (c) across other levels of exposure | Discussion  Limitations paragraph | Fifth, the use of predominantly European ancestry specific data limits generalisability to other ancestries, the analysis should therefore be replicated using data from other ancestries once available. |
|  | **OTHER INFORMATION** |  |  |  |
| 18 | **Funding** | Describe sources of funding and the role of funders in the present study and, if applicable, sources of funding for the databases and original study or studies on which the present study is based | Metadata at publication site | Metadata |
| 19 | **Data and data sharing** | Provide the data used to perform all analyses or report where and how the data can be accessed, and reference these sources in the article. Provide the statistical code needed to reproduce the results in the article, or report whether the code is publicly accessible and if so, where | Metadata at publication site | All data used in this study is available to download freely at cited publications and links, as follows[…] |
| 20 | **Conflicts of Interest** | All authors should declare all potential conflicts of interest | Metadata at publication site | There are no competing interests |

This checklist is copyrighted by the Equator Network under the Creative Commons Attribution 3.0 Unported (CC BY 3.0) license.

1. Skrivankova VW, Richmond RC, Woolf BAR, Yarmolinsky J, Davies NM, Swanson SA, et al. Strengthening the Reporting of Observational Studies in Epidemiology using Mendelian Randomization (STROBE-MR) Statement. JAMA. 2021;under review.

2. Skrivankova VW, Richmond RC, Woolf BAR, Davies NM, Swanson SA, VanderWeele TJ, et al. Strengthening the Reporting of Observational Studies in Epidemiology using Mendelian Randomisation (STROBE-MR): Explanation and Elaboration. BMJ. 2021;375:n2233.
