# Supplementary material for: Multiple anthropometric measures and proarrhythmic 12-lead ECG indices: A mendelian randomization study
Source: PLoS Med. 2023 Aug 8;20(8):e1004275. doi: 10.1371/journal.pmed.1004275 (PMC10443852; doi:10.1371/journal.pmed.1004275)
Supplement: S1 Table — (DOCX) [file pmed.1004275.s002.docx]

S1 Table – Results of multivariable Mendelian randomization (MVMR) analyses. WHR = adjusted waist:hip ratio, BMI = body mass index, b = beta coefficient, se = standard error, pval = p-value, F-stat = conditional F- statistic, CI = Confidence interval, ms = milliseconds. MVMR was performed using the TwoSampleMR package in R^1^. Conditional F-statistics were calculated using the MVMR package in R^2^.

| **Outcome** | **MVMR**  **Variables** | **Exposure** | **nsnp** | **b** | **se** | **pval** | **F-stat** |
| --- | --- | --- | --- | --- | --- | --- | --- |
| **P wave duration** | Height + Fat-free mass + WHR + Fat mass | Height | 38 | 8.349 | 3.372 | 0.013 | 7.70 |
|  |  | Fat-free mass | 15 | -5.064 | 7.805 | 0.516 | 12.35 |
|  |  | WHR | 5 | -1.739 | 4.052 | 0.668 | 6.21 |
|  |  | Fat mass | 5 | 2.815 | 4.269 | 0.510 | 5.36 |
|  | BMI + Height + Fat-free mass + WHR + Fat mass | BMI | 12 | 24.960 | 17.275 | 0.149 | 9.75 |
|  |  | Fat-free mass | 20 | -27.054 | 16.354 | 0.098 | 0.30 |
|  |  | Height | 36 | 20.175 | 9.244 | 0.029 | 0.32 |
|  |  | Fat mass | 11 | -8.986 | 11.737 | 0.444 | 0.35 |
|  |  | WHR | 4 | -1.126 | 3.900 | 0.773 | 0.29 |
|  | Height + Fat-free mass + BMI + Fat mass | BMI | 10 | 23.604 | 16.836 | 0.161 | 0.31 |
|  |  | Fat mass | 9 | -9.867 | 11.608 | 0.395 | 0.36 |
|  |  | Height | 37 | 19.087 | 9.061 | 0.035 | 0.36 |
|  |  | Fat-free mass | 20 | -23.656 | 16.112 | 0.142 | 0.32 |
| **QT interval** | Height + Fat-free mass + WHR + Fat mass | Height | 725 | 0.108 | 0.723 | 0.881 | 11.30 |
|  |  | Fat-free mass | 218 | 1.349 | 1.985 | 0.497 | 3.30 |
|  |  | Fat mass | 35 | 2.552 | 1.246 | 0.041 | 2.99 |
|  |  | WHR | 75 | 0.533 | 0.896 | 0.551 | 4.21 |
|  | BMI + Height + Fat-free mass + WHR + Fat mass | WHR | 70 | 0.288 | 0.805 | 0.721 | 0.48 |
|  |  | BMI | 115 | 4.848 | 2.896 | 0.094 | 10.57 |
|  |  | Fat mass | 67 | -0.471 | 2.111 | 0.824 | 0.58 |
|  |  | Height | 684 | 2.080 | 1.439 | 0.148 | 0.55 |
|  |  | Fat-free mass | 234 | -1.505 | 2.690 | 0.576 | 0.75 |
|  | Height + Fat-free mass + BMI + Fat mass | Height | 737 | 2.567 | 1.417 | 0.070 | 0.48 |
|  |  | BMI | 107 | 5.136 | 2.836 | 0.070 | 0.54 |
|  |  | Fat mass | 62 | -0.070 | 2.076 | 0.973 | 0.58 |
|  |  | Fat-free mass | 241 | -3.040 | 2.662 | 0.253 | 0.72 |

References

1. Hemani G, Zheng J, Elsworth B, et al. The MR-Base platform supports systematic causal inference across the human phenome. *Elife*. 2018;7. doi:10.7554/eLife.34408

2. Sanderson E, Spiller W, Bowden J. Testing and correcting for weak and pleiotropic instruments in two-sample multivariable Mendelian randomization. *Stat Med*. 2021;40(25):5434-5452. doi:10.1002/sim.9133
